# Simulation and mechanism for the Ultrasound-Assisted Oiling-Out Process: A case study using Fructose-1,6-diphosphate

**DOI:** 10.1016/j.ultsonch.2024.106953

**Published:** 2024-06-12

**Authors:** Pengpeng Yang, Qian Wu, Haodong Liu, Shuyang Zhou, Wensu Chen, Huamei Zhong, Keke Zhang, Fengxia Zou, Hanjie Ying

**Affiliations:** a College of Biotechnology and Pharmaceutical Engineering, Nanjing Tech University, Nanjing 211816, China; b Biology+ Joint Research Center, School of Chemical Engineering and Technology, Zhengzhou University, Zhengzhou 450001, China

**Keywords:** Ultrasound, Molecular simulation, Fructose-1, 6-diphosphate, Oiling-out mechanism, Nucleation energy barrier

## Abstract

Liquid–liquid separation, commonly referred to as oiling-out, frequently can occurs during crystallization, especially the anti-solvent crystallization process of phosphoryl compounds, and poses potential hurdle for high-quality product. Efficiently regulating oiling-out during crystallization remains a significant challenge. Among various techniques, ultrasound emerges as a green and effective approach to enhance the crystallization process. However, there is a dearth of in-depth research exploring the microscopic mechanisms of this process. Therefore, our research focused on the fructose-1,6-diphosphate (FDP), a typical phosphoryl compound, to gain a deeper understanding of how ultrasound influences the oiling-out process. The focused beam reflectance measurement (FBRM) technology was used to investigate the oiling-out phenomenon of FDPNa_3_ across various solvent ratios. In addition, the influence of ultrasound on the induction time was studied and the nucleation energy barrier was calculated. Finally, to further unravel the microscopic mechanisms, we utilized molecular simulation techniques to analyze the impact of ultrasound power on the dissolution-precipitation process. Our observations revealed a consistent oiling-out process that attainted a stable state regardless of the solvent employed. Notably, the results of the oiling-out induction time experiments indicated that ultrasound significantly reduced helped lower the nucleation energy barrier of FDP^3-^ ions, thereby dismantling FDP^3-^clusters in solution. Thus, in turn, shortened the reduced induction time and promoted crystallization. Furthermore, ultrasound reduced the interactions between FDP^3-^ions and water molecules as well as FDP^3-^ ions themselves. As simulated field intensity increased, these interaction forces gradually diminished, the thickness of the hydration layer surrounding the FDP^3-^ clusters facilitating the disruption of clusters, ultimately enhancing the crystallization process.

## Highlights

• Influence of ultrasound on the oiling-out process of FDPNa_3_ was investigated.
• Ultrasound destroyed the interaction of FDP-water and hydration layer on surface.
• Ultrasound shortens the induction period for stable phase nucleation.
• Ultrasound lowers the nucleation energy barrier, promoting nucleation of FDP Na_3._

## 1 Introduction

Oiling-out, also known as liquid–liquid separation phenomenon, occurs when solute molecules become concentrated in one phase, resulting in the formation of oil droplet-like new phases owing to disparities in density. This phenomenon commonly arises during the crystallization of proteins [1], small organic compounds, pharmaceutical active ingredients [2], or polymeric systems [3], typically preceding or following nucleation [4]. In industrial production processes, the occurrence of oiling-out often has potential adverse effects on the final product, including severe aggregation, unpredictable particle size, variations, impurity coating, product stability, and polymorphism, ultimately resulting in product quality issues, significant time and economic losses [5]. Therefore, it is imperative to develop a green and effective method to mitigate these challenges.

At the molecular level, substances with a high molecular weight, multiple dissociation sites, complex functional groups, high melting points, and good water solubility are prone to exhibit oiling-out phenomena during anti-solvent crystallization [6]. Recent research has primarily focused on exploring the influence of various factors, including solvent type [7], concentration [8], temperature [9], and impurities [10], on the oiling-out process. There are two distinct types of oil phases: metastable and stable. Our previous study has revealed that a stable oiling-out system of GMPNa_2_ (Disodium Guanosine 5′-Monophosphate) emerges during the secondary nucleation stage, which complicates the crystallization process and results in undesirable crystal properties. Furthermore, the oiling-out process has the potential to yield novel or more stable crystal forms in the metastable oil phase [11]. For instance, Sun et al. [12] observed that oiling-out crystallization resulting in octahedral crystals, whereas conventional cooling crystallization produced lamellar crystals. Subsequent studies research has successfully obtained various spherical crystals by intentional utilization of the oiling-out technique [13].

The double hydrophase, another typical liquid–liquid separation phenomenon, commonly arises in the aqueous system that composed of polymer-salt[14]. This phenomenon primary stems from the salting-out effect, disparities in hydrophilic and hydrophobic properties, as well as the spatial resistance effect. Double hydrophase systems are well established and widely used in various fields. Therefore, when delving into the intricate nature of the oiling-out process, we can draw upon the relevant ideas and methodologies from this system.

The crystallization process is inherently driven by supersaturation [15]. As supersaturation increases, solute molecules collide and aggregate among themselves, ultimately forming clusters when supersaturation reaches a critical level. The nucleation energy barrier plays a pivotal role in this process, as variations in it can significantly impact crystal properties. Once the nucleation energy barriers are surpassed, the intermolecular forces between solute molecules prevail, leading to the formation of stable crystal nuclei and ultimately crystallization[16]. However, an increasing supersaturation often results in the oiling-out phenomenon. Excessively high supersaturation can induce, unpredictable nucleation, making it challenging into regulate the final crystal morphology and particle size. While oiling-out is thermodynamically favorable, it is kinetically disadvantageous. The intricate interplay between the intermolecular forces of solute and solvent molecules creates a complex and variable system. It is necessary to additional energy input to overcome the nucleation energy barriers, disrupt the thermodynamically stable state, and promote the formation of stable crystal nuclei.

Ultrasonication emerges as an “ innovative and green” technique that harnesses the power of cavitation induced by acoustic and hydrodynamic effects to deliver energy [17], [18]. This form of energy aids significantly in overcoming the nucleation energy barriers within a crystallization system, thus fostering a more efficient crystallization process. Prior studies have reported that ultrasound-assisted crystallization can influence crucial aspects such as crystal size [19], polymorphism [20], [21], crystal morphology, induction time, and even the narrowing of the metastable zone [22], [23]. Furthermore, ultrasound facilitates primary nucleation at relatively low degrees of supersaturation and promote secondary nucleation. However, it is noteworthy that the mechanisms underlying the influence of ultrasound on diverse crystallization processes are distinct, necessitating a unified theoretical mechanism. Additionally, there is a scarcity of research on the effects of ultrasound on the oiling-out process, limiting our understanding of the mechanisms involved.

Fructose-1,6-diphosphate (FDP, molecular formula: C_6_H_14_O_12_P_2_, CAS No. 488–69-7, molecular weight: 340.12 g/mol) stands as a typical phosphoryl compound. Its detailed information refers to Fig. 1 in the Supplementary Information. The crystallization of phosphoryl compounds differs significantly from ordinary small organic molecules, posing greater more challenging [24]. Their more and intricate dissociation states, typically higher water solubilities, and stronger solute–solvent interactions, which often results in higher nucleation energy barriers and makes the ordered self-assembly of solute molecules more difficult, thereby hindering favorable nucleation [25], [26]. During our initial experiments, we encountered distinctive oiling-out phenomena during the crystallization of FDPNa_3_. FDP exhibited more complex dissociation behaviors compared to GMPNa_2_, and its liquid–liquid two phases caused by oil-out in anti-solvent crystallization exhibit something similar to the stability of the double hydrophase. However, the revenant research on this kind of oiling-out system remains understudied. Therefore, this study aims to investigating the effects of ultrasound on the oiling-out process in FDPNa_3_ systems via experimental and molecular simulation methodologies. Additionally, we strive to illustrate the underlying mechanism of ultrasound’s impact on the oiling-out process using molecular simulation approaches.

**Fig. 1:**
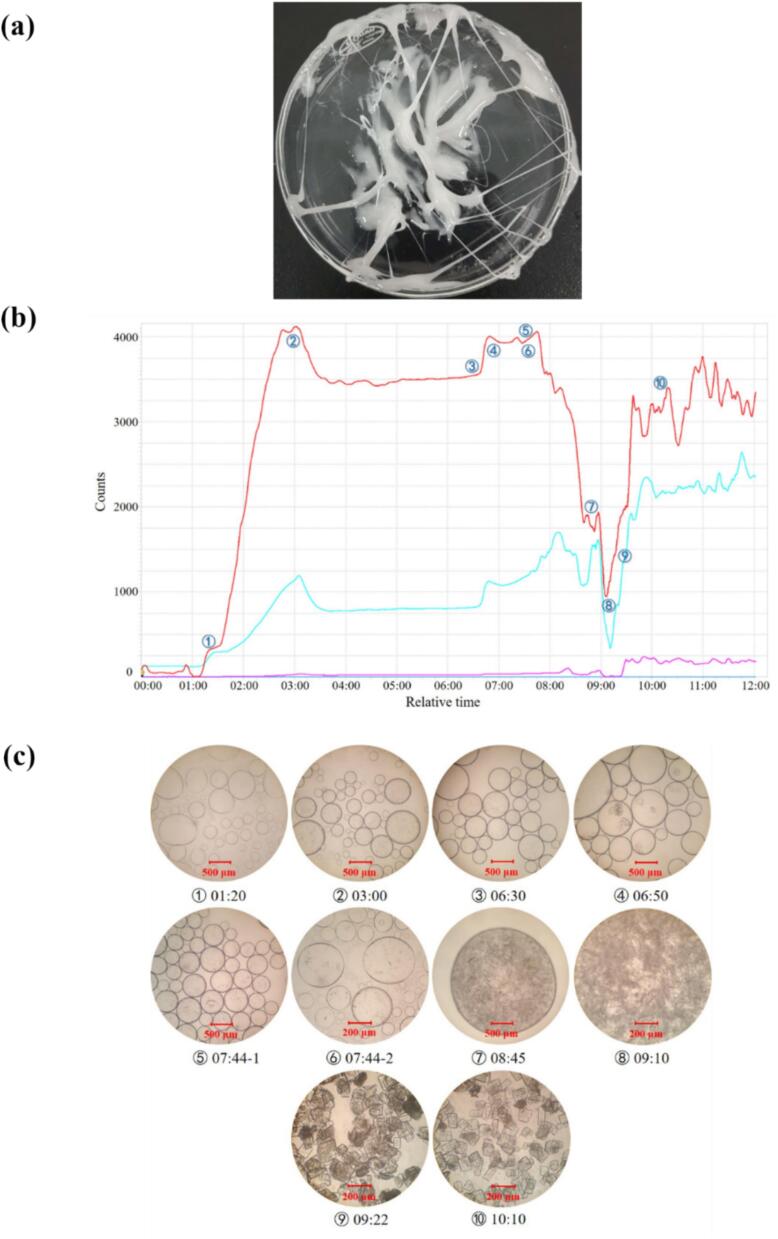
(a) The resulting paste-like state evolved from oil phase that caused by the conventional continuous addition of ethanol into FDPNa_3_ aqueous solution; (b) FBRM curves of the oiling-out crystallization process of FDPNa_3_. Red: chord length, < 10 μm; cyan: chord length, 10–50 μm; violet: chord length, 50–150 μm; blue: chord length, 150–300 μm;(c) Microscopic observation of the system’s state. (For interpretation of the references to colour in this figure legend, the reader is referred to the web version of this article.)

## 2 Materials and methods

### 2.1 Materials

The FDPNa_3_ salt (99 % purity) was supplied by Nanjing Tongkai Zhaoye Biotechnology Co., Ltd. Analytically pure ethanol was obtained from Wuxi City Yasheng Chemical Co., Ltd. All the materials were used without further purification.

### 2.2 Apparatuses

An ultrasonic bath (KH-P15H, Kehao, Nanjing Kehao Intelligent Science and Technology Co., Ltd. Nanjing, China) with an adjustable power range from 0 to 400 W generated a continuous ultrasonic field set at 100 W. For the cooling experiment, a thermostatic water bath (CK-400GD, Xinzhi Biotechnology Co., Ltd., Ningbo, China) was used. An electronic analytical balance (FA3204C, Tianmei Balance & Instruments Co., Ltd.) with an accuracy of ± 0.0001 g was used for all mass measurements. Anti-solvent crystallization experiments involving the dropwise addition of ethanol were performed using an electronic peristaltic pump from Tronray Pumps while constantly stirring the solvent using a suspended stirrer paddle (EUROSTAR 20, IKA, China).

### 2.3 Crystallization process of FDPNa_3_

Briefly, 35 g of FDPNa_3_ was added to 100 mL of ultrapure water at 25 ℃ with constant stirring at 200 r/min. Subsequently, 250 mL of ethanol was slowly added, and the samples were acquired with a pipette for experimental observation. In addition, 35 g of FDPNa_3_ was added to 100 mL of ultrapure water, and then a total of 160 mL of ethanol was pumped in at a rate of 1 mL/min starting at 0.33 h; During the process, the amounts of crystalline species added was 0.1 % by mass of the solute. The signal was monitored using FBRM with continuous viewing to observe the moment of a sudden signal change.

### 2.4 Impact of the solvent on the oiling-out process

Different ethanol concentrations (0.4, 0.6, 0.8, 1.0, 1.2, 1.4, and 1.6 times the volume of water) were investigated during ethanol solubilization. Following oiling-out, the two phases were separated after resting for 2 h. The volumes of the two phases were measured using a graduated cylinder, and the contents of the components in the aqueous and oil phases were measured. The experimental system contained 7 g FDPNa_3_ and 20 mL ultrapure water at 25 ℃ and 200 rpm. The FDP^3-^ ion content in the two phases was determined by titration and calculated according to the molecular weights of the FDP acids. Water content was determined using a Karl Fischer moisture titrator, and ethanol content was determined using calculations. Each measurement was performed three times for each sample, the average value was taken for calculation, and all the final data were expressed as mass fractions (w%). Supersaturation in the two phases was calculated from the measured FDPNa_3_ content and its solubility, σ = (C − C*)/C*, where C and C* are the actual and saturated concentrations, respectively.

### 2.5 Ultrasound-assisted induction time experiments

The application of ultrasound during crystallization promotes cavitation in the solution. The oscillation of the solution at different frequencies improves the mixing conditions at the molecular level, thereby increasing the diffusivity of the solute molecules in the solution and the probability of collision of nucleation sites, which in turn affects the crystallization process [27].

First, 1, 3, 5, 7, and 9 g of FDPNa_3_ were dissolved in 20 mL of water. The FDPNa_3_ solution was prepared in a magnetically stirred water bath at 40 ℃ for 5 min to achieve homogeneity. The peristaltic pump was calibrated to accurately calculate the volume of ethanol added based on the time and flow rate, set to 0.2 mL/min. This equilibrium FDP^3-^ solution was then transferred to a jacket and placed in a 100 W water bath sonicator connected to a thermostatic water bath. The temperature was lowered from 40 to 5, 15, and 30 ℃ at a rate of 0.5 ℃/min, and ethanol was added dropwise until crystallization occurred. Parallel experiments were conducted without sonication under identical conditions.

### 2.6 Effect of ultrasound on the three-phase diagram

Using different concentrations of FDP^3-^ aqueous solutions, ethanol was pumped in at a flow rate of 0.2 mL/min at 5, 15, and 20 ℃ with 200 r/min stirring to prevent sudden clumping of the high-density phase. The process was continuously observed using an optical microscope to capture oiling-out and induction time, and the amount of ethanol pumped was recorded. The experimental conditions for the ultrasound group were consistent with those in Section 2.5.

### 2.7 Molecular dynamics simulation for ultrasound treatment

In this study, all-atom molecular dynamics (MD) simulations were conducted using Gromacs 2018.8 [28] coupled with a general Amber force field (GAFF) [29] and an explicit solvent representation. The GAFF parameters for the solvent molecules were sourced from the Virtual Chemistry database, except for ethanol molecules, which was parameterized according to the standard GAFF procedure. The GAFF ensured that the properties were consistent with the experimental data for all the investigated solvents. Specific values comparing the experimental solvent densities with the obtained solvent parameters were also analyzed.

The.pdb file underwent initial Gaussian processing to acquire more accurate restrained electrostatic potential charges [30]. To simulate the energy impact of the ultrasound field on the solution, a pulsed sinusoidal electric field was applied along the x-axis, as described in principle. The specific solute–solvent ratios used in the ultrasound experiments were converted to molar ratios for consistency. FDP, ethanol, and water molecules were confined within a 5 × 5 × nm^3^ box. Sequential steps of energy minimization, NVT, NPT, and MD were executed, lasting 20, 40, 40, and 40 ns, respectively. All simulations were performed under isothermal–isobaric (NPT) conditions, maintaining a pressure of 1 bar and a temperature of 300 K. The simulations utilized the Berendsen barostat [31] and Bussi-Donadio-Parrinello thermostat [32]. For simulations focusing on the crystal-solution interface, semi-isotropic pressure control was utilized alongside three-dimensional periodic boundary conditions, mirroring the actual crystal-solution interface conditions. A sinusoidal pulsed electric field, as expressed in Eq. (1), simulated the energy effect of the ultrasonic field on the solution.

(1) $$E\left(t\right)=E_{0}exp\left[-\frac{{\left(t-t_{0}\right)}^{2}}{2\sigma^{2}}\right]\cos\left[\omega \left(t-t_{0}\right)\right]$$

Where E_0_ (v/nm) represents the maximum achievable electric field strength in the electric field, σ denotes the duration of the electric field's continuous action time, t_0_ denotes the time at which the electric field reaches its maximum value, and ω = 2πc/λ, which is used to control the frequency of the change.

To analyze the inter-component interaction forces, initially, utilize the “gmx make_ndx” command to group the different components in the box: all solute molecules, all ethanol molecules, and all water molecules. Subsequently, use the “gmx energy” command to select the desired force pairs, such as “solute–solute” and “solute-water”.; The forces calculated by GROMCAS are mainly categorized into the long-range (LR) and short-range (SR) forces. Although 1–4 non-bonding forces as a way to calculate non-bonding forces within molecules are also included between these two force types, for accurate calculations, the 1–4 non-bonding forces are calculated separately. When calculation of inter-component forces, the 1–4 force term is set to 0, and a cut-off distance of 5 nm is chosen, ensuring the entire box for force analysis. At this point, SR represents the accurate total interaction forces. The forces calculation are further divided into coulombic forces (Coul) and van der Waals forces (Vdw). For Vdw the classical Lennard-Jones potential (LJ) method is used. In summary, the inter-component interaction force is calculated as the sum of the short-range Coulomb force Coul-SR and the short-range van der Waals force LJ-SR. Detailed calculations for this section are described in the supplementary information.

The interaction forces between FDP^3-^ − FDP^3-^ calculated in this paper refers to the sum of the intermolecular interaction force and the intramolecular non-bonding interaction force. The forces between the remaining components refer to intermolecular interaction forces. After processing the kinetic equilibrium, the results were visualized using VMD [33] and analyzed with Multiwfn[34].

## 3 Results and discussion

### 3.1 Crystallization of FDPNa_3_

FDPNa_3_ is a hydrophilic phosphoryl compound and the anti-solvent crystallization is considered as its most common crystallization method. We found that adjusting the type of anti-solvent does not eliminate the issue of oiling-out (referring to methanol, ethanol, n-propanol…). Herein, the Ethanol, considered a relatively green and safe organic solvent, was selected, as the anti-solvent of FDPNa_3_ aqueous solution crystallization for exploring of its oil-out process. It was found that the conventional continuous addition of the ethanol failed to crystallize the FDP. Instead, a paste was formed, as shown in Fig. 1(a).

The entire oiling-out and crystallization process was subjected to a comprehensive dynamic and microscopic analysis using FBRM and optical microscopy. The four curves that were obtained showed the variation in the number of oil droplets or crystal particles with different chord length distributions over the crystallization time or the number of crystal particles with the crystallization time. The red curve illustrated the entire process of oiling-out and crystallization.

As shown in Fig. 1(c) ①, the emergence of oil droplets becomes evident after 1 h 20 min into the experiment. Initially, the solution was clear, but over time, it became turbid, aligning with the corresponding change in signal ① in Fig. 1(b). As the ethanol was continuously added, the oil phase volume expands, the oil droplets grow larger, resulting in a significant intensification of the signal. The system’s components gradually transition from an initial homogeneous phase to a final two-phase distribution corresponding to the change of signal ② in Fig. 1(c). At this stage, nucleation has not occurred yet. Due to the decrease in the number of oil droplets and their volume increase, smaller oil droplets coalesce into larger ones, causing the number of oil droplets slightly decrease and stabilize at a certain value. Even after continued stirring and ethanol addition, nucleation is still not observed, as seen in Fig. 1 (c) ③. Subsequently, the addition of 0.5 g of crystal seed leads to a rapid increase in the number of particles within the oil droplets at 6.83 h. Inside the droplets, crystal seed were discernible, as shown in Fig. 1 (c). At 6.83 h, crystals were found to form within the oil droplet, initiating secondary nucleation, as depicted in Fig. 1(c) ⑤ & ⑥. Following nucleation, crystal growth consumes the supersaturation in the system, especially the solutes in the oil phase, leading to a rapidly decrease in the oil phase volume. This also caused the droplet weight to increase, resulting in sedimentation and aggregation as seen in Fig. 1(c) ⑦. As droplets continue to settle towards the bottom and become less susceptible to agitation by the paddle, the FBRM probe has difficulty detecting the clusters at the bottom, leading to a sharp drop in the signal as shown in Fig. 1(c) ⑧. Finally, when the supersaturation consumed during the growth of crystals reaches a critical level, and the difference between the two phases is insufficient to maintain the two − phases state, the oiling-out state is broken and the crystals encapsulated within enter the homogeneous phase. The clustered particles break apart due to stirring, and a portion of the stirred crystals are released into the solution, causing the signal to rise sharply again, as shown in Fig. 1(c)⑨&⑩.

### 3.2 Impact of the solvent on the oiling-out process and composition analysis

**Fig. 2:**
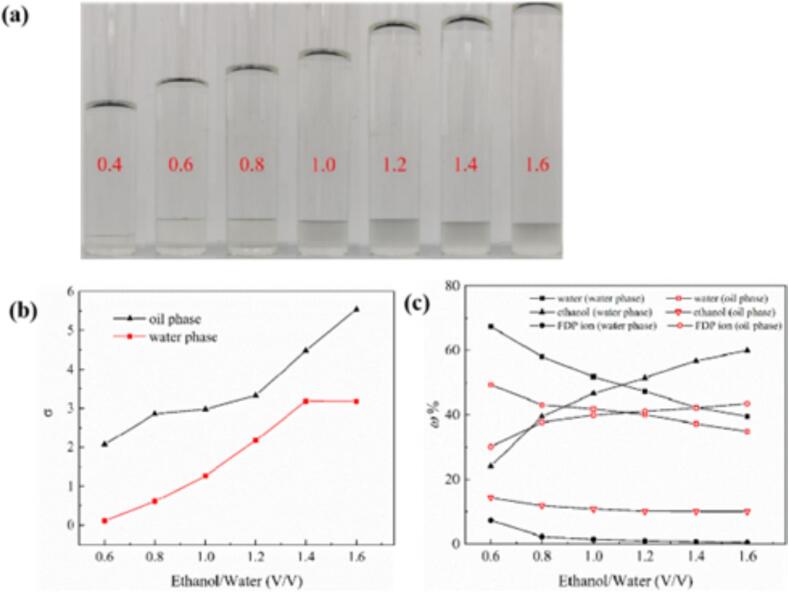
(a) The oiling-out state of FDPNa_3_ corresponding various ethanol–water ratio: from left to right, 0.4 ∼ 1.6 times the volume of water; (b) Supersaturation of water phase and oil phase under various ethanol–water ratio; (c) Proportions of water, FDP, and ethanol in the two phases.

Fig. 2 shows the effect of different ethanol −water ratios on the emergence and evolution of the two distinct phases during the oiling-out process. As shown in Fig. 2(a), increasing ethanol–water ratio initially leads to a corresponding augmentation the volume of the high-density phase (oil phase). This increase culminated in a peak volume at a specific ethanol–water ratio. Beyond this ratio, however, the volume of the high-density phase began to decline, indicating a shift in the system’s equilibrium. Concurrently, the volume of the low-density phase continued to expand, reflecting the redistribution of components within the system as the solvent components was altered.

Fig. 2(b) reveals an interesting trend in the FDP content within the high-density phase as the ethanol–water ratio gradually increased. Although the FDP content gradually rises, it does so at a relatively slower pace. This is attributed to the fact that FDP has low solubility in ethanol; however, as more ethanol was introduced, it enriched the oil phase, thereby gradually increasing the FDP content in the high-density phase. Conversely, as the ethanol ratio decreased, the ethanol tends to accumulate in the low-density phase, resulting in a higher ethanol content. Simultaneously, the water ratio decreased because of the stronger interaction between FDP and water. In addition, the natural affinity between water and ethanol led to the gradual migration of water molecules to the low-density phase during ethanol addition.

Moreover, as shown in Fig. 2(b), in the high-density phase, rapid supersaturation occurred owing to FDP enrichment and water loss. Conversely, in the low-density phase, the initial low FDP content and continuous ethanol addition caused an initial increase in supersaturation, followed by stability after reaching a certain threshold.

It is worth noting that supersaturation alone is insufficient promote favorable nucleation and crystallization of FDP. External interference or stimuli are necessary to trigger the crystallization process effectively.

### 3.3 Ultrasound-assisted induction time experiments

When measuring the induction time of FDPNa_3_, it is necessary to ensure that all samples underwent continuously stirred; otherwise, it would remain in an excessively prolonged steady state (more than 96 h), hindering the observation of nucleation and impeding the accurate measurement of the induction time. The relevant calculation method for induction time is presented in Supplementary information.

**Fig. 3:**
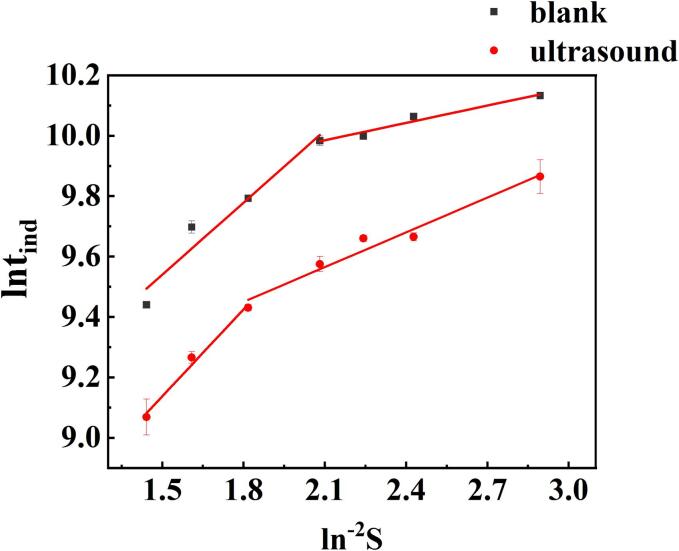
Effect of ultrasound on the induction time of FDPNa_3_.

Upon the addition of ultrasound (Fig. 3), a marked reduction in the induction time was observed compared to the blank group, which indicated that the 100 W ultrasound water bath played a vital role in promoting nucleation.

The induction time data was used to calculate a series of nucleation parameters, including the nucleation energy barrier. As shown in Table 1, an increase in supersaturation led to a corresponding decrement in the induction time, revealing a two-stage induction time with two distinct slopes, which was indicative of a discontinuous stage in the data. Additionally, as supersaturation increased, the nucleation energy barrier decreased. However, the nucleation energy barrier significantly exceeded that of common substances. This substantial barrier may be the primary reason for the oiling-out of FDPNa_3_.

**Table 1:** CNT parameters (γ, ΔGc, and rc) for FDPNa_3_ nucleation at different supersaturation (S) levels and 100 W ultrasound treatment.

S	Time(s)	lnt_ind_	γ(mJ/m^2^)	Nc	ΔG_c_(J/mol)	r_c_ (nm)
1.8	19,800	9.893	5.552	5.636	2743.888	0.44
1.9	15,840	9.670	5.552	4.328	2301.089	0.41
1.95	15,840	9.670	5.552	3.843	2125.567	0.39
2	14,400	9.574	5.552	3.437	1973.126	0.37
2.1	12,600	9.441	7.234	6.199	3809.599	0.46
2.2	10,800	9.287	7.234	5.166	3373.318	0.43
2.3	9000	9.105	7.234	4.382	3022.863	0.41

Comparing Table 1, Table 2, it can be found that the nucleation energy barrier of FDPNa_3_ is considerable and decreases with the increase of supersaturation. The high nucleation energy barrier may be the reason for the phenomenon of oiling-out occurring without nucleation following solute clustering. The effect of ultrasonication on the crystallization of oiling-out is mainly reflected in the shortening of the induction period, though interestingly, ultrasonication also tends to increase the nucleation energy barrier of FDPNa_3_. In the study of Parimaladevi[35],it was observed that ultrasound significantly shorten the metastable zone. However, there are limited studies focusing on nucleation parameters, and the underlying mechanism of ultrasound’s effect remains rarely involved. Notably, while ultrasound increased the nucleation energy barrier in the experiments, it does not mean that ultrasound inhibits nucleation. Nucleation is a complex process influenced by numerous factors, and the nucleation energy barrier is merely one of them. An increased rate of nucleation can also promote the formation of crystallography. The influence of ultrasound on nucleation can be analyzed from two perspectives. From a thermodynamic standpoint, ultrasonic vibration may induce local heating, causing energy changes. Though this change may be negligible for the general system, for the metastable oil phase, the disappearance of local oil droplets releases a significant amount of solutes into the aqueous phase, resulting in an increase in local supersaturation. From the kinetic perspective, ultrasonic vibration reduces the collision distance between local solute molecules, thereby accelerating collision-induced nucleation.

**Table 2:** CNT parameters (γ, ΔGc, and rc) for FDPNa_3_ nucleation at different supersaturation (S) levels without ultrasound treatment.

S	Time(s)	lnt_ind_	γ(mJ/m^2^)	Nc	ΔG_c_(J/mol)	r_c_ (nm)
1.8	25,200	10.134	4.578	3.160	1538.40	0.36
1.9	23,400	10.060	4.578	2.427	1290.14	0.33
1.95	21,960	9.996	4.578	2.1.55	1191.73	0.32
2	21,600	9.980	4.578	1.927	1106.27	0.31
2.1	18,000	9.798	6.963	5.530	3398.02	0.44
2.2	16,200	9.692	6.963	4.608	3008.87	0.41
2.3	12,600	9.441	6.963	3.909	2696.28	0.39

### 3.4 Effect of ultrasound on the three-phase diagram

The temperature variations did not effectively accelerate the crystallization process of FDPNa_3_. however, the effect of ultrasound on FDPNa_3_ crystallization varied at different temperatures.

**Fig. 4:**
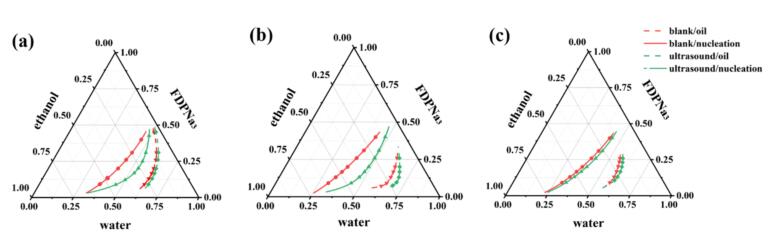
Effects of ultrasound on liquid–liquid separation and nucleation curves at different temperatures: (a) 5 ℃, (b) 15 ℃, and (c) 20 ℃.

As shown in Fig. 4, the oiling-out and nucleation curves influenced by the 100 W continuous ultrasound water bath displayed a more pronounced forward shift compared to the blank group. This result indicated the significant influence of ultrasound on oiling-out crystallization. However, measuring the oiling-out curves was challenging owing to the rapid oiling-out observed in high-concentration FDP^3-^ solutions following ultrasound application of ultrasound. This rapid oiling-out led to a diminished gap between the oiling-out and nucleation curves, thereby facilitating the entire process.

Given the variance in supersaturation at different temperatures, the oiling-out curves exhibited minimal changes. However, the nucleation curves underwent a notable forward shift at 5 and 15 ℃, whereas no significant shift was observed at 20 ℃. This suggests that, at this temperature, the supersaturation might have been relatively low, rendering the role of ultrasound-assisted nucleation less pronounced.

### 3.5 Molecular simulation analysis

**Fig. 5:**
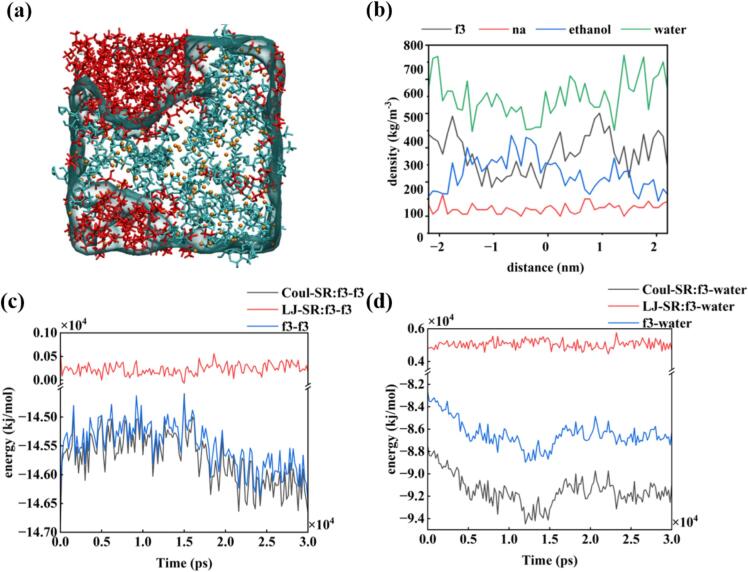
Molecular dynamics simulation of FDPNa_3_ oiling-out process in ethanol–water ratio (1:7) at 288.15 K. (a) Last frame of simulation. (b) Density distribution of the last frame. (c) Interaction forces of FDP^3-^ − FDP^3-^ during the MD simulation.; (d) Interaction forces of FDP^3-^-water during the MD simulation.

As shown in Fig. 5(a), a clear split phase was observed, with ethanol aggregated in one phase while the solute and water were distributed in the other phase. Moreover, Na ions were heavily aggregated in the vicinity of the solute owing to electrostatic effects. As shown in the density diagram in Fig. 5(b), the density distribution was not homogeneous because ethanol and the solute were distributed in both phases. Fig. 5(c) and (d) show that the interaction force between solutes was larger than that between the solutes and water, and the interaction force between solutes gradually decrease over time. The final interaction force reached approximately −146000 kJ/mol, which was beneficial for the nucleation process. However, there were numerous dissociation sites, the state of the phosphoric acid group was complex, and the interaction force between solutes was stable and strong, instead of forming a sub-stable state. The difference in the interaction forces shown in Fig. 5(c) and (d) might be the reason for the generation of these two phases.

**Fig. 6:**
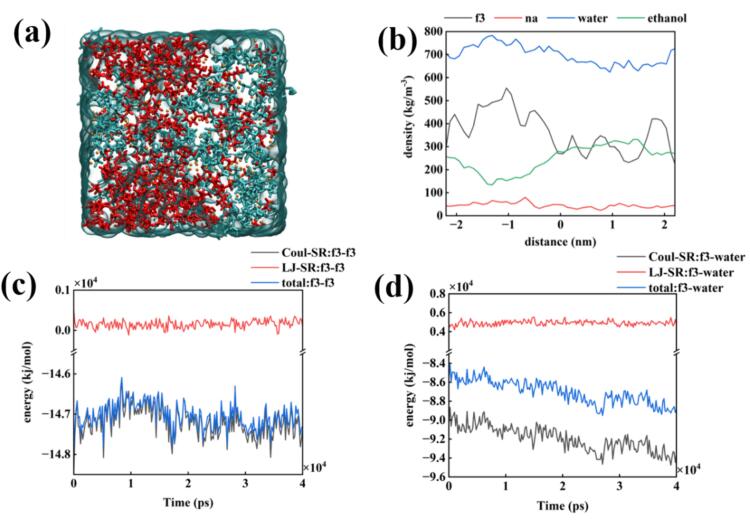
Molecular dynamics simulation of FDPNa_3_ introduced by sinusoidal electric field with a maximum value of 15 V/nm in the x-axis direction; (a) Last frame of the simulation; (b) Density distribution of the last frame; (c) Interaction forces of FDP^3-^–FDP^3-^ during the MD simulation; (d) Interaction forces of FDP^3-^-water during the MD simulation.

Comparing Fig. 6(a) with 5(a), the delamination of the two phases following the addition of ultrasound was less obvious than before. Ethanol was still aggregated, and the solute was slightly aggregated, which demonstrated that ultrasound played a certain role in alleviating the clustering. However, the presence of strong interactions still produced the clustering phenomenon. The density distribution was different because ethanol functioned as a poor solvent for FDPNa_3_. When analyzed from the molecular view, ethanol and FDP^3-^ had a weak interaction, rendering density distribution unavoidable. As shown in Fig. 6(c) and (d), The solute interaction force fluctuates more due to the ultrasonic field, which becomes larger in comparison to Fig. 5. The applied pulsed electric field might be small, which needs to be further confirmed.

**Fig. 7:**
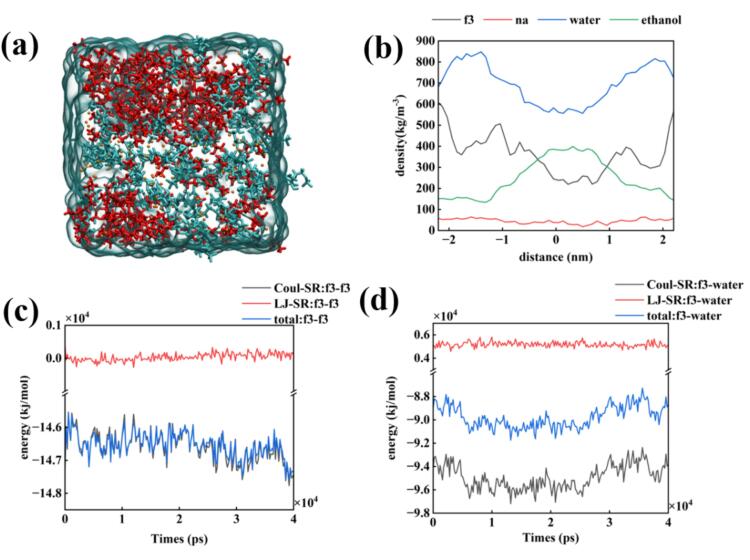
Molecular dynamics simulation of FDPNa_3_ introduced by sinusoidal electric field with a maximum value of 20 V/nm in the x-axis direction; (a) Last frame of the molecular dynamics simulation; (b) Density distribution of the last frame; (c) Interaction forces of FDP^3-^ − FDP^3-^ during the MD simulation; (d) Interaction forces of FDP^3-^-water during the MD simulation.

**Fig. 8:**
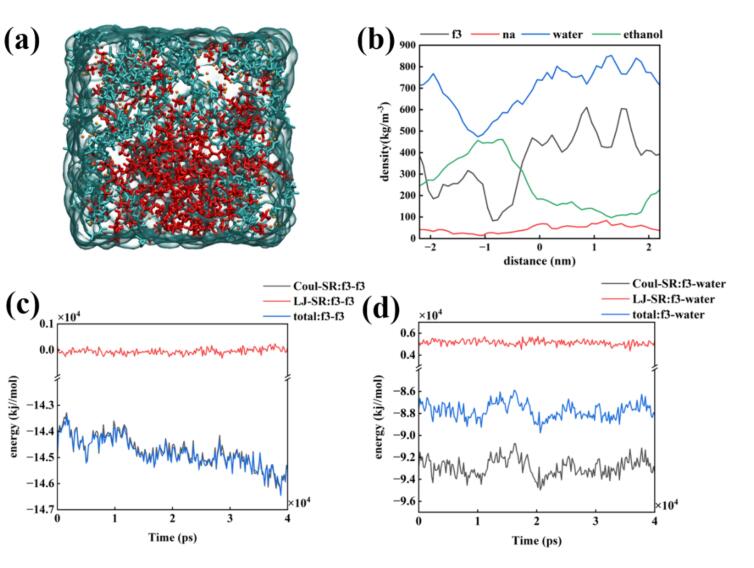
Molecular dynamics simulation of FDPNa_3_ introduced by sinusoidal electric field with a maximum value of 25 V/nm in the x-axis direction. (a) Last frame of the simulation; (b) Density distribution of the last frame; (c) Interaction forces of FDP^3-^-FDP^3-^ during the MD simulation; (d) Interaction forces of FDP^3-^-water during the MD simulation.

As shown in Figs. 7(a) and 8(a), the degree of clustering of ethanol decreased with an increase in simulated field strength, indicating that the addition of ultrasound had a significant effect on increasing molecular collisions. The density map exhibited a decrease in the tightness of the two types of clusters. The FDP^3-^ − FDP^3-^ interaction force for the first 20 ns shows a decreasing and then increasing trend, however, after ultrasound stopped, all interactions tended to increase. Moreover, the difference between the FDP^3 -^- FDP^3-^ and FDP^3-^-water interaction forces slightly decreased, which may be the reason for the disappearance of the two phases.

Based on the quantified interaction forces and density diagram results, ultrasound affected the molecular motion and intermolecular forces by providing energy to the solution, resulting in different molecular states.

### 3.6 Mechanism of the ultrasound-assisted oiling-out process

**Fig. 9:**
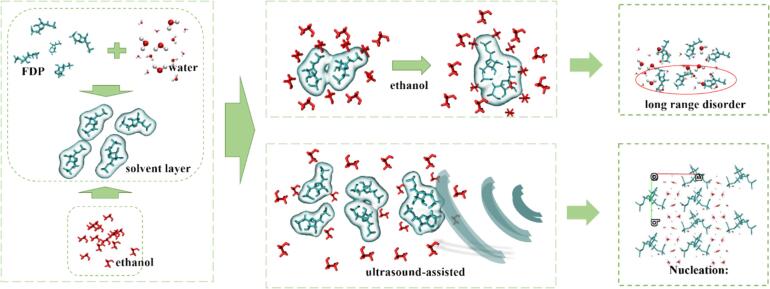
Schematic diagram of the oiling-out mechanism.

Fig. 9 schematically illustrates the ultrasonic action mechanism of oiling-out crystallization investigated in this experiment. In an aqueous solution, FDPNa_3_ exhibits high solubility due to its dissociation into the −3 valence state and subsequent formation a robust hydration layer rich in hydrogen bonding sites. This hydration layer effectively shields the FDP^3-^ molecules from direct contact with each other.

Upon the addition of the anti-solvent ethanol, its compatibility with water and competing interaction forces cause ethanol molecules to displace a part of water molecules around hydrated FDP^3-^ species. As ethanol, is a poor solvent for FDPNa_3_, it gradually decreases the solubility of FDPNa_3_ in water, leading to an increase in the degree of supersaturation. As the addition of ethanol introduced, the solvent layer comprising water molecules around FDP^3-^ molecules weakens, and the bonding between FDP^3-^ and water becomes weaker, which allows FDP^3-^ molecules to come into closer contact, overcome the nucleation energy barrier, and initiate nucleate. In conventional crystallization experiments, increasing the degree of supersaturation, typically drives nucleation. However, although FDP^3-^ had a high tendency to nucleate under high supersaturation conditions, the strong force between FDP^3-^ and water molecules in the hydrated layer often prevented rapid nucleation, even when supersaturation was achieved. Continuous ethanol addition alone may not be effective in increasing supersaturation.

At a certain degree of supersaturation, the introduction of continuous ultrasound could disrupt the hydration layer surrounding the FDP^3-^, which significantly increases the probability of solute collision and nucleation events. As a result, supersaturation became more effective, facilitating rapid nucleation. Moreover, the introduction of ultrasound could provide an external energy, assisting the FDPs in surmounting the high nucleation energy barrier and promoting molecular assembly.

## 4 Conclusion

In this study, we delved into the oiling-out process of FDPNa_3_, specifically, examining the significant role of ultrasound in enhancing this process from experimental and molecular simulation perspectives. Ultrasound, as a green and efficient method, has the potential to improve the products quality caused by oiling-out in industrial crystallization, shorten the production cycle, avoid the excessive addition of anti-solvents. The oiling-out phenomenon in FDPNa_3_ was conspicuously observed using FBRM technology across various solvent ratios. As the solvent content increased, the oil and the water phases attained a dynamic equilibrium state. The results of the oiling-out induction time experiment indicated that ultrasound effectively accelerated the nucleation rate of FDPNa_3_, disrupting the solute clusters in solution and thus shortening the induction time. The crystallization mechanism of FDPNa_3_ closely adhered to the two-step nucleation theory, which involves initial cluster formation, followed by rearrangement into crystals. Furthermore, molecular simulations offered quantitative insights into the intermolecular forces and ordering within the system. Ultrasound diminished the interactions between FDP^3-^-water and FDP^3^-FDP^3-^, with the interaction forces gradually weakening as the simulated field intensity increased. This led to a reduction in the thickness of the hydration layer surrounding the FDPNa_3_ clusters, thereby facilitating their disruption.

To sum up, this study provides a novel approach and perspective for addressing oiling-out issues in industrial crystallization. By adjusting the amount of solvent and ultrasound parameters, the oiling-out process can be optimized to achieve a more favorable dynamic equilibrium state, ultimately enhancing product quality. This work lays the foundation for the broader application of ultrasound in oiling-out process, providing effective solutions for industrial production.

## CRediT authorship contribution statement

**Pengpeng Yang:** Writing – review & editing, Supervision, Software, Resources, Project administration, Methodology, Investigation, Funding acquisition, Conceptualization. **Qian Wu:** Writing – review & editing, Writing – original draft, Methodology, Investigation, Formal analysis, Data curation, Conceptualization. **Haodong Liu:** Project administration, Methodology, Investigation, Conceptualization. **Shuyang Zhou:** Investigation. **Wensu Chen:** Methodology, Data curation. **Huamei Zhong:** Methodology, Data curation. **Keke Zhang:** Project administration, Methodology, Investigation. **Fengxia Zou:** Visualization, Project administration, Methodology, Funding acquisition. **Hanjie Ying:** Supervision, Software, Resources, Methodology, Funding acquisition, Conceptualization.

## Declaration of competing interest

The authors declare that they have no known competing financial interests or personal relationships that could have appeared to influence the work reported in this paper.
