# Supplementary material for: Simulation and mechanism for the Ultrasound-Assisted Oiling-Out Process: A case study using Fructose-1,6-diphosphate
Source: Ultrason Sonochem. 2024 Jun 12;108:106953. doi: 10.1016/j.ultsonch.2024.106953 (PMC11228588; doi:10.1016/j.ultsonch.2024.106953)
Supplement: Supplementary Data 1 [file mmc1.docx]

**Supporting Information**

Simulation and Mechanism for the Ultrasound-Assisted Oiling-Out Process: A Case Study using Fructose-1,6-diphosphate

Pengpeng Yang^a^, Qian Wu^a^, Haodong Liu^a^, Shuyang Zhou^a^, Wensu Chen^a^, Huamei Zhong^a^, Keke Zhang^b^, Fengxia Zou^a,*^ ,Hanjie Ying^a^

^a^ College of Biotechnology and Pharmaceutical Engineering, Nanjing Tech University, Nanjing 211816, China

^b^ Biology+ Joint Research Center, School of Chemical Engineering and Technology, Zhengzhou University, Zhengzhou 450001, China

Corresponding Author Email: Fengxia Zou, zoufengxia@njtech.edu.cn

**1. Force field selection**

Three force fields suitable for small molecule force field were mainly screened, and the rest might be more suitable for the simulation of biological macromolecules. The reference value of force field selection was selected as RMSD for comparison. The main method was to use Guassian to minimize the energy of solute molecules, and take the optimization results of quantum chemistry as the input structure of molecular dynamics, and then take a value of RMSD.

Opls_aa

Root mean square deviation after lsq fit =0.04322 nm

gromos_54a7
Root mean square deviation after lsq fit =0.05704 nm
gaff
Root mean square deviation after lsq fit = 0.02452 nm

**2. The calculation of induction time**

The induction period is an important parameter in a theoretical study, which is important for the elaboration of the nucleation mechanism and the understanding of the nucleation process. It can also calculate a series of parameters in the nucleation process, such as solid-liquid phase surface tension, critical particle size, critical nucleation molecular number, and the metalstable zone of crystallization can also be obtained by extrapolation.

The induction time is proportional to the inverse of the nucleation rate, and the classical primary homogeneous nucleation theory for spherical crystals is described below.

Assuming a spherical shape[1], the derivation leads to the basic equation for the stationary nucleation rate J:

$J=Aexp\left( -\frac{\Delta G_{c}}{kT} \right)=Aexp(-\frac{16\pi\sigma^{3}{v_{m}}^{2}}{3k^{3}T^{3}{ln}^{2}S})$ (1)

where A is the pre-exponential factor, ΔGc is the activation Gibbs energy of nucleation. k is the Boltzmann constant, T is nucleation temperature, σ is the solid−liquid interfacial energy, υm is the solute molecular volume, and S is the supersaturation. The thermodynamic driving force for nucleation is the difference in chemical potential between the solute in solution and in the crystalline bulk phase, Δμ. Neglecting the concentration dependence of the activity coefficient gives.

$\Delta\mu\approx kTlnS=kTln\frac{x}{x^{*}}$ (2)

where x and x* are actual and equilibrium solute mole fractions, respectively. The chemical potential is a partial molar term, and the interfacial energy is the Gibbs energy of the interface, and accordingly the derivation inherently includes all contributions to changes in the Gibbs energy of the system. The nucleus is defined as a crystalline particle of a critical size, i.e., sufficient for growth to be thermodynamically favorable. Its radius depends on both the interfacial energy and the nucleation driving force according to

$r_{c}=\frac{2v_{m}\sigma}{\Delta\mu}$ (3)

The number of molecules making up the nucleus, n_c_, is estimated using the radius and the molecular volume:

$n_{c}=\frac{4\pi{r_{c}}^{3}}{3v_{m}}$ (4)

The induction time, t_ind_, is the time period from the establishment of the supersaturated state to the first observation of crystals in the solution, and it is usually assumed that the induction time is inversely proportional to the nucleation rate and the volume.

$lnt_{ind}=-lnJV=-lnAV+\frac{B}{T^{3}{ln}^{2}S}$ (5)

Induction time experiments are usually evaluated by plotting tind vs. T^−3^ ln^−2^ S, allowing the determination of the interfacial energy from the slope B:

$B=\frac{16\pi\sigma^{3}{v_{m}}^{3}}{3k^{3}}$ (6)

Knowing the interfacial energy allows for calculation of the radius of the critical nucleus and the nucleation work, ΔGc = 4πrc 2 /σ.

**3. The detailed calculations for interaction forces**

We checked the definition of the unit of calculation of “gmx energy" command in official website of GORMACS, to make sure that the "mol" here is the mol of the component (60 FDP^3-^ molecules), not the mol of a single molecule.

First, allow us to provide a detailed explanation of the steps involved in the calculation of the interaction energy, after completing the molecular dynamics simulations:

a) Define the index file; classify all molecules into solute, ethanol, water, and Na^+^ and name them f3, eth, water, and na in that order.

b) Modify the .mdp file; define the energy groups as components, specifically “ energy groups” = f3 eth na water”.

c) Rerun molecular dynamics simulation

e) Use “ gmx energy -f md.edr” for energy analysis.

Then we choose the components that we want to calculate, if we choose Coul-SR: f3-eth and LJ-SR: f3-eth, the result is the interaction energy between the component with the name of f3 and the component with the name of eth. because the two groups don't contain the same molecules, so at this point, the interaction force is completely the intermolecular interaction energy. If we choose Coul:SR-f3-f3 and LJ-SR: f3-f3, because the two groups also contain the same molecules, so the calculated energy at this point is the sum of the intermolecular interaction energy and intramolecular interaction energy. At this point, “kJ/mol” is actually “kJ/60mol ", so the energy at this time is very large.

By simulating some simple systems, using the same method and the same parameters as above, with the difference of the number and type of molecules in the system. We hope to prove that there is no problem in the logic of the calculation after detailed consideration and verification in this way, and on the other hand, we can borrow a simpler example to prove the scientific validity of the numerical values.

The first simple system: there are 2 ethanol molecules and 4049 water molecules in the system, and all the rest of the conditions and implementation are the same as in the manuscript.

a) We divide the two ethanol molecules into one group named “e”. The sum of Coul-SR: e-e and LJ-SR: e-e is 76 kJ/mol;

b) Using another grouping method, the two ethanol molecules are divided into two groups and named “e1”, “e2”. As shown in Fig. 1, the energy between “e1” and “e1” is almost half of that in a). This is in agreement with our conclusions at the beginning, that the interaction energy between the same components, calculated by this method, contains two parts. And the real intermolecular interaction energy should be the sum of Coul-SR: e1-e2 and LJ-SR: e1-e2, from the “Average” column in Fig. S1, this value is very small, because ethanol and water are compatible, and two ethanol molecules are far away from each other in water, so the intermolecular interaction energy is small.


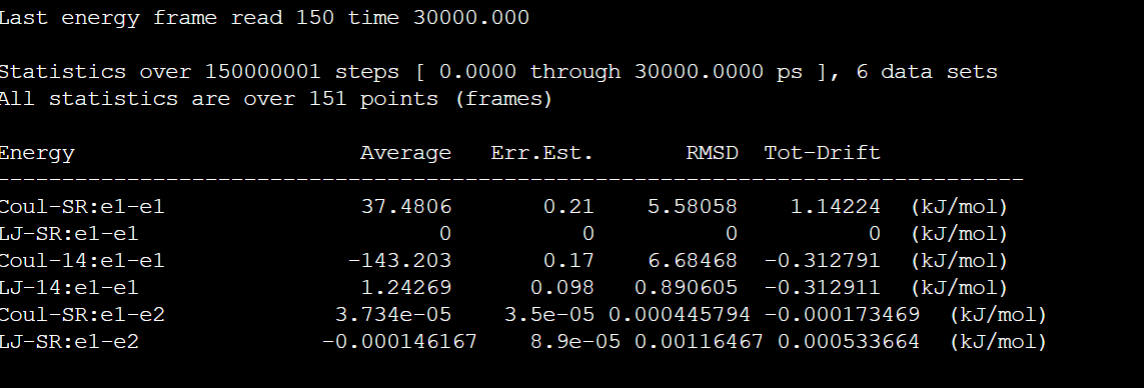


Fig. S1 Energy calculations for the simple system ethanol-water

The second simple system: there are two FDP^3-^, six Na^+^ ions, and 3981 water molecules in the system, and all the rest of the conditions and implementation methods are the same as in the Marked revised manuscript. The two FDP^3-^ molecules were divided into two different components named f1 and f2, all the water molecules were divided into one group named water, and all the Na^+^ ions were divided into one group and named Na. The results are shown in Fig. S2. According to our previous presentation, the sum of the Coul-SR: f1-f1 and the LJ-SR: f1-f1 is the intramolecular nonbonding of the group f1 interactions, this value is -2471.85 kJ/mol, and the sum of Coul-SR: f2-f2 and LJ-SR: f2-f2 is -2417.41 kJ/mol, which are almost equal. We can assume that the intramolecular nonbonding interaction energy of a single FDP^3-^molecule in the system of this paper is about equal to -2471 kJ/mol. The smaller sum of Coul-SR: f1-f2 and LJ-SR: f1-f2 is due to the small number of molecules and the large intermolecular distance.


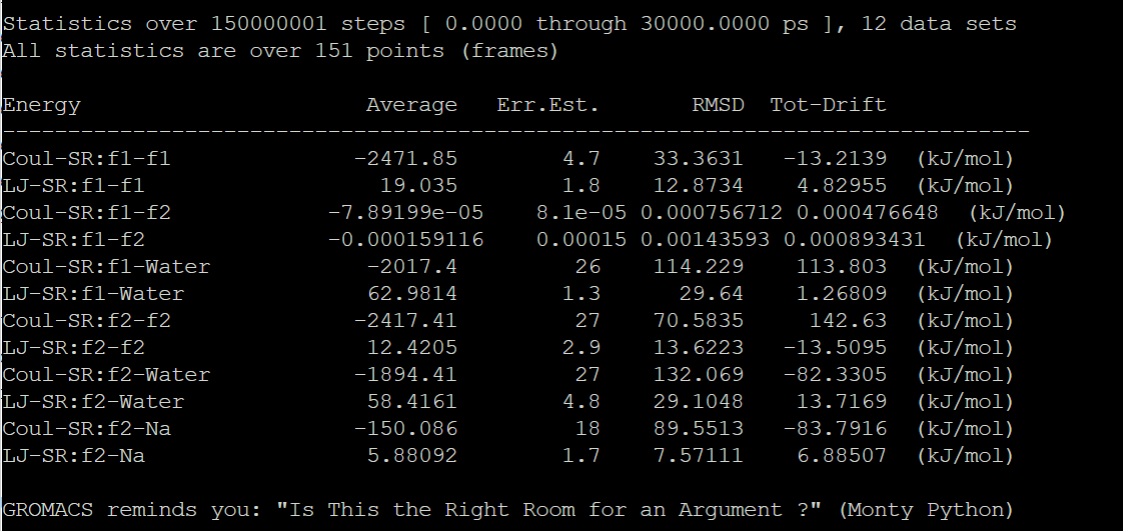


Fig. S2 Energy calculations for the simple system FDP^3-^ aqueous solution

Using the example of the two simple systems above, we randomly select 30 FDP^3-^molecules out of 60 FDP^3-^molecules, divide them into 30 groups. Then we calculated the intramolecular interaction energy separately, getting the average value of the FDP^3-^molecular intramolecular interaction energy, and then use -146000 - (-2471 x 60) = 2260 kJ/mol. 2260 kJ/mol is the 60 mol of FDP^3-^ intermolecular interaction energy.

Considering the phenomenon of cluster aggregation in our simulated system, it is normal that the solute molecules have small spacing and large intermolecular interaction energies. Moreover, we mainly study the trend of the interaction energy before and after the addition of ultrasonic field, and the trend is still the same after the subtraction of the intramolecular non-bonding interaction energy. In “Molecular Design and Characterization of Ionic Monomers with Varying Ion Pair Interaction Energies” and “Benchmark Calculations of Interaction Energies in Noncovalent Complexes and Their Applications", there are similar examples of calculated interaction energies to prove that our calculated values are within reasonable limits.

The interaction energy in this paper includes inter- and intramolecular non-bonding interactions and state that all interactions in the paper that refer to FDP^3-^ - FDP^3-^ all refer to the sum of the two.

[1] H. Yang, M. Svärd, J. Zeglinski, Å.C. Rasmuson, Influence of Solvent and Solid-State Structure on Nucleation of Parabens, Crystal Growth & Design, 14 (2014) 3890-3902.dx.doi.org/10.1021/cg500449d
